# 1200V 4H-SiC MOSFET with a High-K Source Gate for Improving Third-Quadrant and High Frequency Figure of Merit Performance

**DOI:** 10.3390/mi16050508

**Published:** 2025-04-27

**Authors:** Mingyue Li, Zhaofeng Qiu, Tianci Li, Yi Kang, Shan Lu, Xiarong Hu

**Affiliations:** 1 School of Electrical Engineering, Southwest Jiaotong University, Chengdu 611756, China; limingyue_62@163.com (M.L.); zfq2002@my.swjtu.edu.cn (Z.Q.); 2024210806@my.swjtu.edu.cn (T.L.); yikang@my.swjtu.edu.cn (Y.K.); mountainlu@my.swjtu.edu.cn (S.L.); 2 Key Laboratory of High Performance Scientific Computation, Xihua University, Chengdu 610039, China

**Keywords:** high-k, source-gate, SiC, MOSFET

## Abstract

This paper proposes a 1200V 4H-SiC MOSFET incorporating a High-K dielectric-integrated fused source-gate (HKSG) structure, engineered to concurrently enhance the third-quadrant operation and high-frequency figure of merit (HF-FOM). The High-K dielectric enhances the electric field effect, reducing the threshold voltage of the source-gate. As a result, the reverse conduction voltage drops from 2.79 V (body diode) to 1.53 V, and the bipolar degradation is eliminated. Moreover, by incorporating a shielding area within the merged source-gate architecture, the gate-to-drain capacitance *C*_gd_ of the HKSG-MOS is reduced. The simulation results show that the HF-FOM *C*_gd_ × *R*_on,sp_ and *Q*_gd_ × *R*_on,sp_ of the HKSG-MOS are decreased by 48.1% and 58.9%, respectively, compared with that of conventional SiC MOSFET. The improved performances make the proposed SiC MOSFEET have great potential in high-frequency power applications.

## 1. Introduction

Silicon carbide (SiC) MOSFETs have emerged as prominent power devices due to their superior characteristics, including a wide bandgap, high critical breakdown electric field, high current density, and excellent thermal conductivity. These attributes surpass traditional silicon materials, making SiC MOSFETs ideal for applications in automobiles, intelligent power systems, national defense, and aerospace [1,2,3,4].

In power electronics, the conventional use of anti-parallel diodes to sustain inductor currents persists, but the direct use of SiC MOSFETs’ parasitic body diode risks bipolar degradation, impacting on-resistance and blocking capabilities [5,6]. To elevate power density and mitigate this, researchers are exploring innovations within SiC MOSFET cells. Studies [1,7,8,9] propose integrating Schottky Barrier Diodes (SBDs) into SiC MOSFETs, offering cost, space, and efficiency benefits. However, the semiconductor–metal interface may have molecular discontinuities, leading to irregularities, defects, and leakage currents under strong electric fields. Alternatively, research [10,11,12,13] introduces SiC MOSFETs with Low-Barrier Diodes (LBDs), enabling low-voltage reverse conduction due to channel depletion. To further optimize performance, an LBD combined with a source-gate (SG) structure has emerged [12,14]. The SG [15,16] structure significantly improves the third-quadrant performance of the device by forming an inversion layer, while its gate-shielding effect further optimizes the high-frequency figure of merit (HF-FOM). To enhance dynamic characteristics such as HF-FOM, researchers have also explored heterojunction-based approaches [17,18]. Nevertheless, these methods introduce interfacial defects and reliability concerns due to lattice mismatch and high interface trap densities. Both strategies—SG and heterojunction designs—suffer from intricate fabrication processes and elevated costs, limiting their practical implementation.

This paper proposes a fused source gate integrated with High-K dielectric (HKSG) MOSFET structure [19,20,21,22,23]. This structure employs a simplified source-gate design to control the N-type channel, incorporating a high-k dielectric material as the gate dielectric to optimize third-quadrant characteristics. When the source gate is connected, it effectively shields one side of the gate’s electric field. As a result, the effective facing area of the gate oxide capacitance is reduced, which, in turn, decreases the gate-to-drain capacitance (*C*_gd_). This combined design not only decreases the device’s capacitance but also significantly cuts down the high-frequency figure of merit *C*_gd_ × *R*_on,sp_ and *Q*_gd_ × *R*_on,sp_. Compared with traditional multi-gate structures, it shows remarkable advantages.

## 2. Device Structure and Mechanism

**Figure 1:**
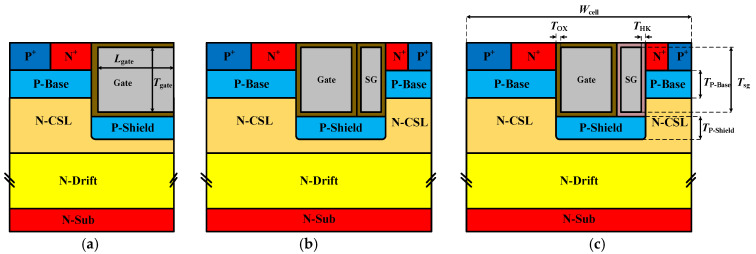
Schematic cross-sectional view of (**a**) Con-MOS, (**b**) SG-MOS, and (**c**) HKSG-MOS.

Figure 1a–c present the schematic cross-sectional configurations of the conventional SiC MOSFET (CON-MOS), the source-gate MOSFET (SG-MOS) and the proposed High-K source-gate MOSFET (HKSG-MOS), respectively. As shown in Figure 1c, the HKSG-MOS introduces an additional channel on the right side of the device. The channel is controlled by the source gate. Moreover, the silica dielectric surrounding the source gate is replaced by the high-k material. The incorporation of the high-k dielectric significantly lowers the threshold voltage compared to the turn-on voltage of the MOS parasitic diode.

**Figure 2:**
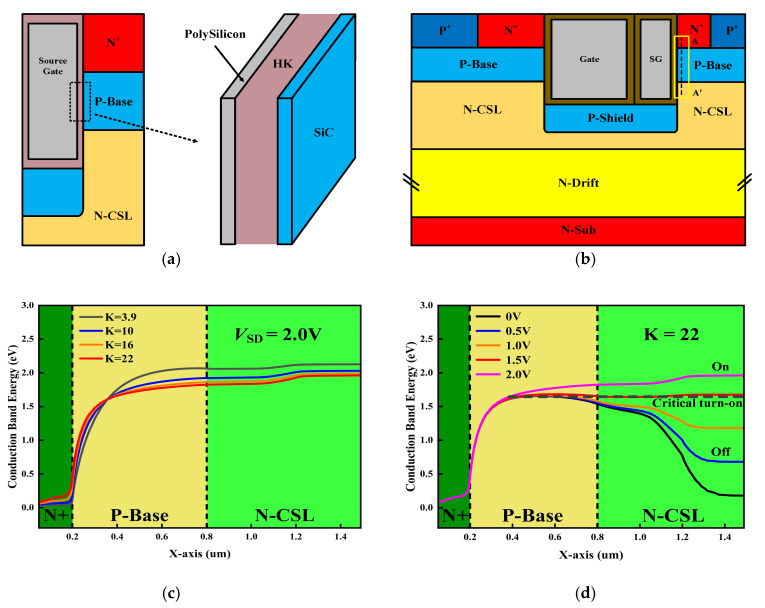
Schematic view of (**a**) the simplified mezzanine plate capacitors. (**b**) Cross-sectional view along with line A–A′. (**c**) Conduction band energy distribution along A–A′ cut-line at different K values. (**d**) Conduction band energy distribution along A–A′ cut-line at different *V*_SD_ values.

To better understand the impact of the SG on reverse conduction characteristics, the structure can be simplified into a metal–insulator–semiconductor (MIS) parallel plate capacitor, as shown in Figure 2a. As the dielectric constant of the material increases, the capacitance of the parallel plate capacitor will increase, allowing more electrons to accumulate under the same voltage, thereby lowering the threshold voltage. Figure 2b shows the cross-sectional view of the proposed structure with the line A-A′. To understand the operating mechanism of the device during reverse conduction, it is crucial to clarify the conduction band energy distribution in the region where line A-A′ is located. Figure 2c shows the conduction band energy (*E*_C_) along line A-A′ with different K values at *V*_SD_ = 2.0 V. As can be seen in Figure 2c, the *E*_C_ decreases with an increase in K value. As a result, the reverse conduction voltage is decreased. Figure 2d shows *E*_C_ along line A–A′ with different source-to-drain voltages (*V*_SD_) when K = 22. The *E*_C_ at the N-CSL region increases with sn increase in *V*_SD_. Once the electron energy is higher than the potential barrier (*V*_SD_ = 1.5 V), the electrons in the N-CSL region could flow through the P-Base to the N^+^ region. Therefore, unipolar conduction in the MOS channel begins, which eliminates bipolar degradation and improves the reliability of the device.

In order to investigate the characteristics of the proposed structure, 2D numerical simulations were performed by Sentaurus TCAD 2022 [24]. The physical models in the simulation mainly include Aniso (Mobility Valanche), effective intrinsic density (Old Slotboom), mobility (Incomplete Ionization High-Field-Saturation Enormal), and recombination (Shockley–Read–Hall Doping Dep Auger Avalanche). Detailed parameters used in the simulation are shown in Table 1.

## 3. Simulation Results and Discussion

**Figure 3:**
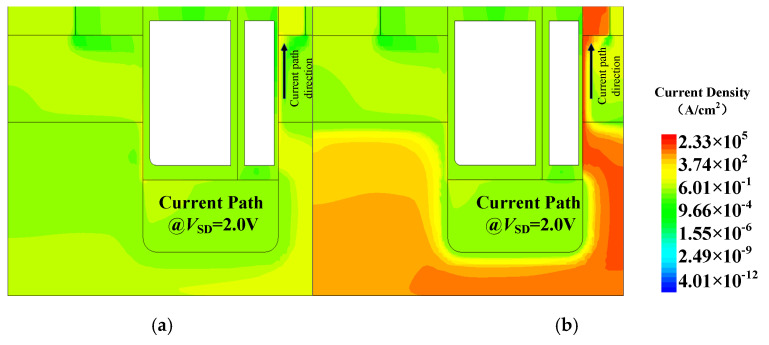
Schematic diagram of reverse conduction current path: (**a**) SG-MOS; (**b**) HKSG-MOS.

**Figure 4:**
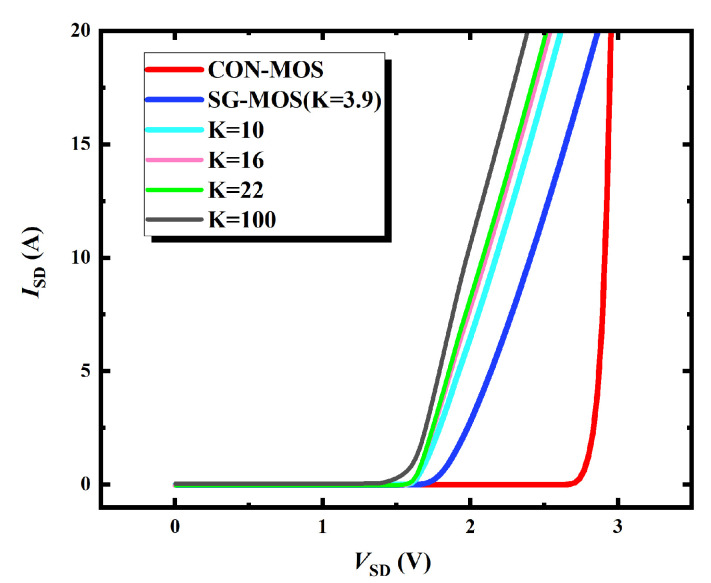
Reverse conduction I-V curves of CON-MOS, SG-MOS, and HKSG-MOS.

Figure 3 gives the reverse conduction characteristics of SG-MOS and HKSG-MOS at *V*_SD_ = 3 V. Figure 3 indicates that the third-quadrant conduction current predominantly concentrates beneath the right-side SG, thereby effectively suppressing the body diode activation and avoiding bipolar degradation. Moreover, due to the introduced high-k dielectric, the reverse conduction voltage of HKSG-MOS is reduced compared with that of SG-MOS. Figure 4 shows that, when the K values increase from 3.9 to 100, the reverse conduction voltage decreases from 1.65 V to 1.41 V. More than that, the enhanced electron concentration induced by the HKSG leads to a significant increase in the third-quadrant current. As can be seen from Figure 4, the reverse current increases from 2.75 A (K = 3.9) to 10.51 A (K = 100) at *V*_SD_ = 2.0 V. Figure 4 also indicates that, due to third-quadrant unipolar conduction, the reverse *V*_on_ of HKSG-MOS is decreased by 49.46% compared with that of CON-MOS.

**Figure 5:**
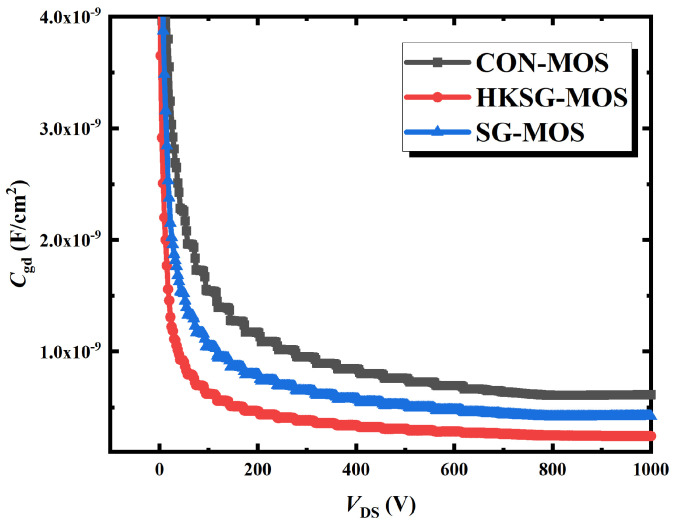
The gate-to-drain capacitance C-V characteristics of CON-MOS, SG-MOS, and HKSG-MOS.

**Figure 6:**
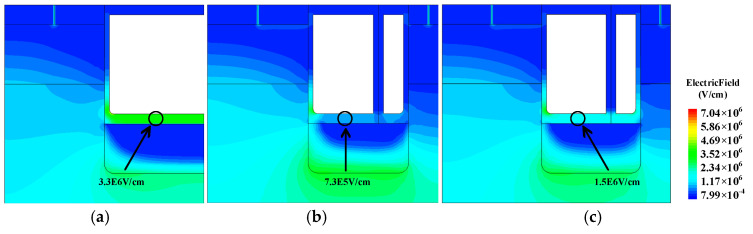
Electric field distributions of (**a**) CON-MOS, (**b**) SG-MOS, and (**c**) HKSG-MOS.

The gate-to-drain capacitance (*C*_gd_) of conventional MOS, SG-MOS, and HKSG-MOS are illustrated in Figure 5. The *C*_gd_ extracted at *V*_DS_ = 800 V is 607.84, 415.75, and 248.02 pF/cm^2^ for CON-MOS, SG-MOS, and HKSG-MOS, respectively. Because of the weakened electric field distribution in the dielectric (labeled in Figure 6), the *C*_gd_ of the HKSG-MOS is reduced. Figure 6 shows the 2D electric field distributions of CON-MOS, SG-MOS, and HKSG-MOS. As can be seen in Figure 6, a portion of the gate-directed electric field lines are redistributed to the source-side SG, thereby reducing the *C*_gd_, with this shielding effect becoming more pronounced at higher K values. Therefore, the *C*_gd_ of the HKSG-MOS is the smallest among these structures.

**Figure 7:**
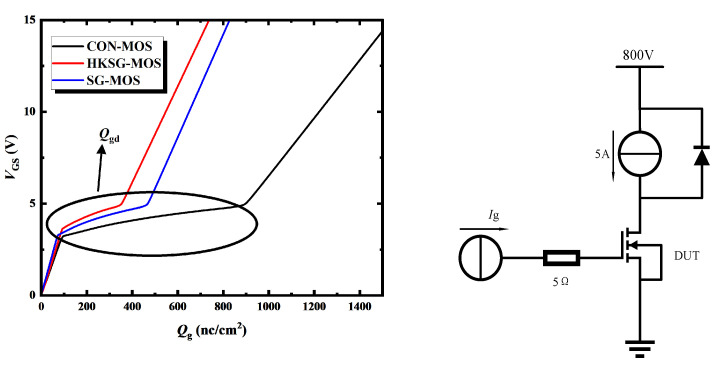
Gate charge characteristics of CON-MOS, SG-MOS, and HKSG-MOS.

Figure 7 shows the gate charge characteristics of CON-MOS, SG-MOS, and HKSG-MOS. As with the reduction in the *C*_gd_, the *Q*_gd_ of HKSG-MOS is reduced by 67.71% and 34.67%, respectively, compared to the CON-MOS and SG-MOS. Consequently, owing to the significantly reduced *C*_gd_ and *Q*_gd_, the high-frequency figures of merit HF-FOM1 (*R*_on,sp_ × *C*_gd_) of the HKSG-MOS are reduced by 48.15% and 34.23% compared to CON-MOS and SG-MOS, respectively. The high-frequency figures of merit HF-FOM2 (*R*_on,sp_ × *Q*_gd_) of the HKSG-MOS are reduced by 58.97% and 27.98% compared to CON-MOS and SG-MOS, respectively.

**Figure 8:**
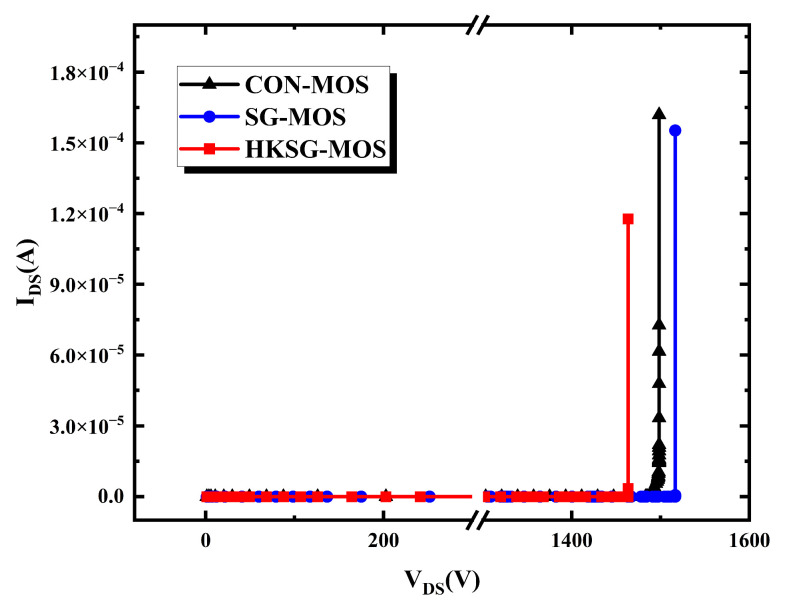
The forward blocking characteristic of CON-MOS, SG-MOS, and HKSG-MOS.

As depicted in Figure 8, the breakdown voltages (*BVs*) of the CON-MOS, SG-MOS, and HKSG-MOS are 1498 V, 1516 V, and 1463 V, respectively. The slight differences in the *BV* of the three structures are due to the varying K value of the SG dielectric, which slightly affects the electric field strength in the right corner of the P-Shield region.

**Figure 9:**
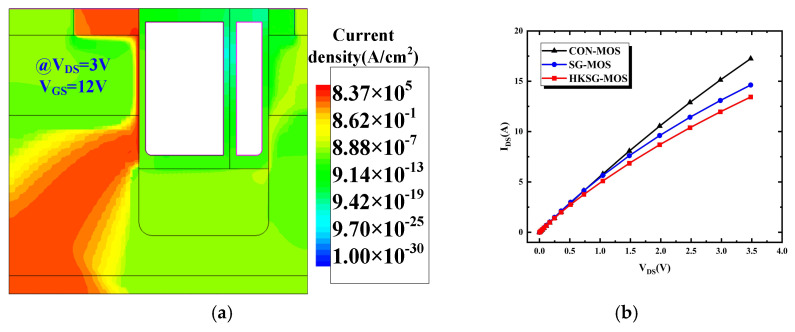
Schematic view of (**a**) the forward conduction current distributions of the HKSG-MOS and (**b**) the forward I–V curves of CON-MOS, SG-MOS, and HKSG-MOS.

Figure 9 shows the forward conduction characteristic of CON-MOS, SG-MOS, and HKSG-MOS. Figure 9a indicates that the forward conduction current predominantly concentrates beneath the gate at the left side of HKSG-MOS. Owing to the modulated bulk electric field by the SG, the width of the current path is narrowed, resulting in an increased *R*_on,sp_ for HKSG-MOS. Figure 9b shows the forward conduction I–V curves of CON-MOS, SG-MOS, and HKSG-MOS at *V*_GS_ = 12 V. When *V*_DS_ = 1 V, the calculated specific on-resistance (*R*_on,sp_) values for CON-MOS, SG-MOS, and HKSG-MOS are 1.44 mΩ·cm^2^, 1.66 mΩ·cm^2^, and 1.83 mΩ·cm^2^, respectively.

**Figure 10:**
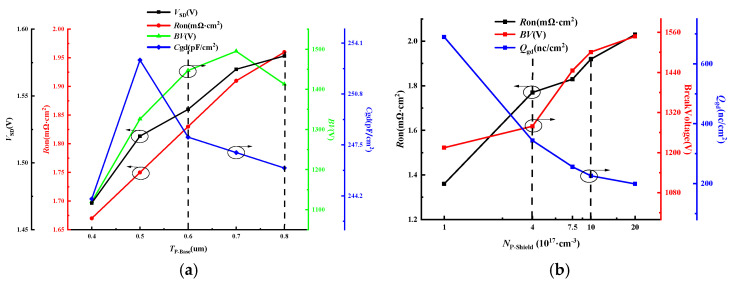
Influence of the P-base depth (*T*_P-Base_) and the concentration of the P-shield (*N*_P-Shield_) on the HKSG-MOS. (**a**) the P-base depth *T*_P-Base_. (**b**) the concentration of the P-shield *N*_P-Shield_.

Figure 10 shows the influence of the P-base depth (*T*_P-Base_) and the concentration of the P-shield (*N*_P-Shield_) on the HKSG-MOS. While the *T*_P-Base_ is increasing, the *C*_gd_ will increase first for the increasement of the electric field besides the oxide gate and finally reduced for the decrease in the effective capacitor area. Meanwhile, the *BV* will increase for the longer channel and finally decrease for the increasement of the electric field. Both of the *V*_SD_ and the *R*_on_ will increase for the channel length. The *N*_P-Shield_ mainly controls the electric field distribution around the gate and the channel. As a result, the enhanced JFET effect increases the *R*_on_ and improves the BV. The *Q*_gd_ is reduced by the electric field shielding. Table 2 compares the main characteristics of CON-MOS, SG-MOS, and HKSG-MOS.

**Figure 11:**
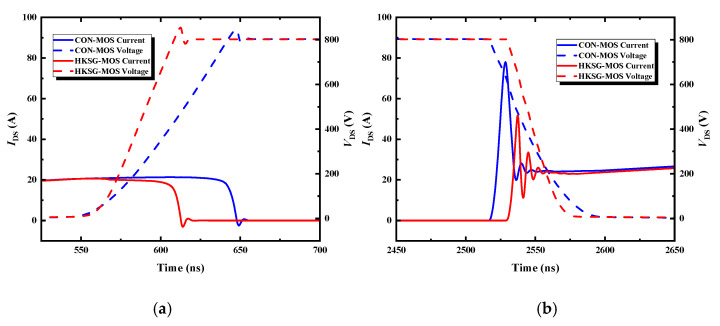
Switch characteristics of HKSG-MOS and Con-MOS. (**a**) Turn-off process. (**b**) Turn-off process.

Figure 11 shows the switch characteristics of HKSG-MOS and Con-MOS. Due to the decrease in the *C*_gd_, the *dv*/*dt* of HKSG-MOS is sharper than Con-MOS. The increase in *C*_gs_ leads to an earlier turning on of Con-MOS. The unipolar reverse conduction model reduces the reverse recovery current, which improves the turn-on loss by 50.7% (HKSG-MOS: 3.56 mJ/cm^2^; Con-MOS: 7.22 mJ/cm^2^). The turn-off loss of HKSG-MOS and Con-MOS are 3.05 mJ/cm^2^ and 5.47 mJ/cm^2^, respectively. The lower *C*_gd_ of HKSG-MOS makes the turn-off process faster and reduces the loss.

**Figure 12:**
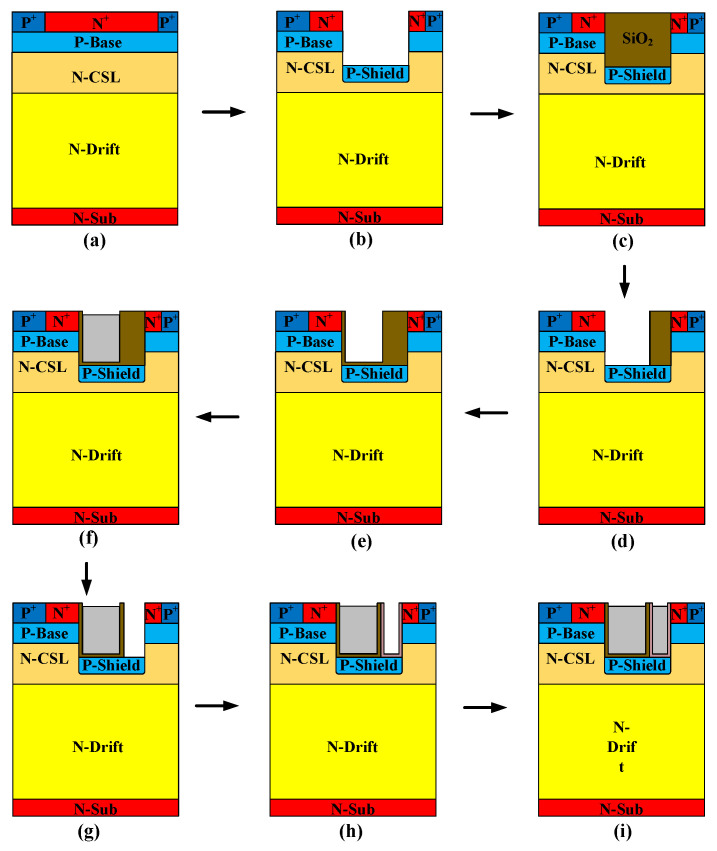
Brief fabrication process flow of HKSG-MOS. (**a**) Epitaxial growth of N-CSL and ion implantation of P-base, P^+^ and N^+^ region. (**b**) Trench etching and P-shield implantation. (**c**) SiO_2_ deposition. (**d**) Gate etching. (**e**) Gate oxidation. (**f**) Poly silicon deposition. (**g**) HK gate etching. (**h**) HK formation. (**i**) Poly silicon deposition.

The process flow is illustrated in Figure 12. Considering the isolation issue between the gate and HKSG, a two-step etching scheme was adopted in this paper to fabricate the trench gate. First, deposit SiO_2_ to form the isolation between the gate and HKSG. Second, etch the gate trench and form the gate oxide by oxidation. After the gate fabrication process, the second etch process is adopted to form a HKSG trench, which leaves a thick enough SiO_2_ wall to isolate the gate and HKSG.

## 4. Conclusions

A 1200V 4H-SiC MOSFET incorporating a High-K dielectric-integrated fused source-gate (HKSG) structure is proposed in this paper. The results demonstrate that the HKSG-MOS achieves a substantially lower reverse conduction voltage (1.41 V vs. 2.79 V) while simultaneously providing a unipolar current path. Notably, by incorporating a shielding area within the merged source-gate architecture, the HFFOM *C*_gd_ × *R*_on,sp_ and *Q*_gd_ × *R*_on,sp_ of the HKSG-MOS are decreased by 48.1% and 58.9%, respectively, compared with that of conventional SiC MOSFET. These findings conclusively establish HKSG-MOS as a highly competitive candidate for high-power and high-frequency applications.

## Figures and Tables

**Table 1:** Detailed parameters used in simulation.

Device Parameters	Value
P-Base depth *T*_P-Base_	0.6 µm
P-Shield depth *T*_P-Shield_	0.5 µm
Gate oxide thickness *T*_OX_	50 nm
Source gate medium thickness *T*_HK_	50 nm
Width of cell pitch *W*_Cell_	2.25 µm
P-Shield doping concentration *N*_P-Shield_	7.5 × 10^17^ cm^−3^
P-Base doping concentration *N*_P-Bsae_	1 × 10^17^ cm^−3^
N-CSL doping concentration *N*_CSL_	2 × 10^16^ cm^−3^
N-Drift doping concentration *N*_Drift_	7.5 × 10^15^ cm^−3^

**Table 2:** Simulated device electrical parameters.

Parameters	CON-MOS	SG-MOS	HKSG-MOS
*V*_SD_ (V)	2.79	1.65	1.53
*BV* (V)	1498	1516	1463
*R*_on,sp_ (mΩ·cm^2^)	1.44	1.66	1.83
*C*_gd_ × *R*_on,sp_ (mΩ·pF)	875.29	690.15	453.88
*Q*_gd_ × *R*_on,sp_ (mΩ·nC)	1143.19	651.28	469.03

## Data Availability

The original contributions presented in the study are included in the article, further inquiries can be directed to the corresponding author.
